# Increasing our knowledge about the epidemiology of *Helicobacter pylori* in Nunavik’s Inuit population (Québec, Canada) using *Qanuilirpitaa?* 2017 cross-sectional survey

**DOI:** 10.1080/22423982.2024.2398864

**Published:** 2024-09-16

**Authors:** Julie Ducrocq, Benoit Lévesque, Gaston De Serres, Véronique Boiteau, Cedric P. Yansouni, Jean-François Proulx, Denis Talbot

**Affiliations:** a Département de médecine sociale et préventive, Université Laval, Québec, Québec, Canada; b Axe Santé des populations et pratiques optimales en santé, Centre Hospitalier Universitaire de Québec, Québec, Québec, Canada; c Direction de la santé environnementale et de la toxicologie, Institut national de santé publique du Québec, Québec, Québec, Canada; d Axe Maladies infectieuses et immunitaires, Centre Hospitalier Universitaire de Québec, Québec, Québec, Canada; e Direction des risques biologiques et de la santé au travail, Institut national de santé publique du Québec, Québec, Québec, Canada; f Bureau d’information et d’études en santé des populations, Institut national de santé publique du Québec, Québec, Québec, Canada; g Department of Medicine, J.D. MacLean Centre for Tropical Diseases, McGill University Health Centre, Montreal, Québec, Canada; h Division of Infectious Diseases and Medical Microbiology, McGill University Health Center, Montréal, Québec, Canada; i Nunavik Regional Board of Health and Social Service, Kuujjuaq, Québec, Canada

**Keywords:** Nunavik, epidemiology, Canada, *Helicobacter pylori*, Inuit, prevalence, Québec

## Abstract

*Helicobacter pylori* is a bacterium that may colonise and proliferate in human stomachs, leading invariably to chronic inflammation and, to a lesser extent, to peptic ulcers and cancer. The main objective of this study is to describe the epidemiology surrounding *H. pylori* in Nunavik’s Inuit population using the 2004 and 2017 Health Surveys. Estimated prevalences were 70.9% for bacterial colonisation using a stool antigens test (SAT), 72.5% for anti-*H. pylori* antibodies, 12.7% for faecal occult blood in participants aged ≥ 50 and respectively of 28.4%, 11.2% and 2.4% for a prior diagnosis of colonisation, gastritis and peptic ulcer in the medical charts, with under five cases of gastric cancer reported. Variables associated with higher SAT+ prevalence were the number of household members (prevalence ratio [PR] = 1.03) and age (quadratic relationship), whereas mainly drinking municipal (PR = 0.84) and natural water (PR = 0.72) compared to bottled water, and increasing alcohol consumption (PR = 0.96) were associated with reduced prevalence. Despite current regional guidelines targeting high risk individuals in the context of high prevalence, Nunavik’s health authorities must remain vigilant by following gastric cancer incidence and the rapid evolution of guidelines, while considering local realities.

## Introduction

### Background

*Helicobacter pylori* is a flagellated bacterium that may colonise and proliferate in the mucus layer of the stomach and the proximal duodenum [1]. While around 70–80% of carriers show no symptoms, its presence invariably leads to chronic inflammation of the gastric mucosa, which can vary in severity and potentially progress to dyspepsia, peptic (a synonym of gastro-duodenal) ulcers or even cancer [2]. Despite literature supporting *H. pylori* as a commensal and/or symbiont bacterium [3], the latest International Maastricht VI/Florence Consensus Report (2022) reflects that *H. pylori* is also robustly demonstrated to be a causal agent of disease states including *H. pylori*-associated gastritis, gastro-duodenal ulcer disease, and some malignancies [4].

According to a recent systematic review and meta-regression, it is estimated that 43.9% of the world’s adult population was infected with *H. pylori* between 2015 and 2022, marking a statistically significant decrease from the estimated global prevalence of 52.6% between 1980 and 1990 [5]. The decline is mainly attributed to the use of antibiotics and the improvement of socioeconomic status, living and hygiene practices (e.g. better water quality supply and wastewater management), but other factors may also contribute [5,6]. For children and adolescents, the global burden of *H. pylori* remained relatively stable (i.e. not statistically different) across periods with prevalence ranging from 22.7% (1995–1999) to 35.1% (2015–2022) [5].

Though humans can acquire other *Helicobacter* species, *H. pylori* remains’ the most frequently reported [7], but its acquisition modes and transmission pathways are incompletely understood. The bacteria’s genetic material has been identified in human saliva, dental plaque, vomitus, and stool, as well as in environmental (i.e. water, soil), food (i.e. milk, vegetables and meat) and animal (i.e. insects, animal tissues and dejections) samples [8–12]. Much epidemiological data supports the predominant human-to human and foodborne transmission routes, through faecal-oral, oral-oral and gastric-oral contacts [7,13], with acquisition occurring at an early stage of life [14]. This hypothesis is supported by similar *H. pylori* genetic exchanges and strains markers within infected individuals sharing the same household [15]. Evidence also supports environmental and occupational transmission pathways that exist through contaminated water, contacts with animals and iatrogenic routes (e.g. through improperly disinfected endoscopes) [13,16].

In the literature, a wide variety of variables have been correlated to *H. pylori* prevalence: age, gender, race/ethnicity, country/region/community, cultural practices (e.g. food sharing), housing conditions (e.g. overcrowding, number of household members), socioeconomic factors (e.g. income and education levels, access to healthcare, social deprivation index, Human Development Index ranking), clinical (e.g. presence of symptoms, past diagnosis or treatment, general condition/comorbidity), individual habits (e.g. diet, smoking, drinking alcohol), water supply (e.g. untreated surface water, end-point water treatment), animal ownership and/or occupational exposure (e.g. exposed to soils) [5,17–20].

Current international guidelines for *H. pylori* management (i.e. Maastricht VI/Florence consensus) consist of using the test-and-treat strategy for uninvestigated young (~ under 50 years) dyspeptic patients, using non-invasive diagnostic tools such as the urea-breath test or stool antigen test (SAT) [4]. Some clinical presentations (e.g. presence of risk factors such as ~ over 50 years) may warrant more advanced investigations such as endoscopy with biopsies of lesions [4]. Treatment options recommended by the International and North American (Toronto Consensus) guidelines consist of prescribing an acid suppressant (i.e. proton-pump inhibitor) in combination with various antibiotics and/or bismuth, with careful attention to regional antibiotic resistance patterns [4,21].

Applying those recommendations to regions of high *H. pylori* endemicity (prevalence > 60%), which is the case with most Arctic indigenous communities, was questioned by specialists working with those populations. In 2016, a group of international experts concluded that, in regions of high prevalence, only dyspeptic patients reporting worrisome systemic symptoms (e.g. weight loss, faecal blood) or dyspeptic patients without improvement following an empirical acid reducing treatment, should be investigated directly by endoscopy [22]. Their justification was based on local native realities (i.e. high probability of re-colonisation, antimicrobial resistance development and weak evidence supporting a benefit of “testing and treating” in indigenous dyspeptic patients). Suspecting that *H. pylori* colonisation was highly prevalent in Nunavik, these “Arctic recommendations” were adapted for Nunavik in 2016, with additional surveillance of Nunavimmiut with a family history of gastric cancer and criteria for performing the Urea Breath Test (UBT) [23]. The *Qanuilirpitaa* 2017 (Q2017) Steering Committee, in consultation with Nunavik medical doctors, highlighted the need to include this pathogen in the gastro-intestinal component of the Nunavik Health Survey to evaluate its burden and fill some gaps of knowledge. Nunavik doctors were concerned by the increasing number of patients colonised with *H. pylori* and have sought formal expertise to assess the prevalence of this infection and the associated risk factors, to eventually better inform management protocols and our understanding of the net benefits and harms of “search and destroy” strategies recommended in guidelines targeting low-endemicity settings.

### Objectives

The main objective of this manuscript is to describe the epidemiology of *H. pylori* in the Quebec Inuit population using the *Qanuippitaa?* (Q2004) and Q2017 Health Surveys. The specific objectives are to: 1) estimate *H. pylori* seroprevalence, the prevalence of colonisation and of at least one prior diagnosis within the medical charts; 2) identify variables associated with these three *H. pylori* primary outcomes; 3) estimate associations between potential clinical consequences (i.e. faecal occult blood, anaemia, weight loss, fresh or digested blood in stools, at least one prior diagnosis of colonisation gastritis, peptic ulcer and gastric cancer in the medical chart), and *H. pylori* serology and colonisation prevalences; and 4) compare serology and SAT results.

## Materials and methods

This manuscript follows the STROBE guidelines (STrengthening the Reporting of OBservational studies in Epidemiology) guidelines [24].

### Setting and participants

Approximately 13 000 Inuit live across the fourteen coastal communities of Nunavik, the administrative region of Quebec (Canada) situated above the 55^th^ parallel. These communities are also separated into two administrative areas: 1) the Hudson coast, situated in the western part of the province and encompassing the villages of Inukjuak, Kuujjuarapik, Puvirnituq, Umiujaq, Akulivik, Ivujivik and Salluit and 2) The Ungava Coast, situated in the northern part, encompassing the villages of Kangiqsujuaq, Quaqtaq, Kangiqsualujjuaq, Kangirsuk, Tasiujaq, Kuujjuaq and Aupaluk). The main objective of the Q2017 cross-sectional survey was to establish a health portrait of Nunavimmiut (people living in Nunavik), which was held from 21 August to 5 October 2017 [25,26]. The Q2017 Nunavik Inuit Health Survey methodological reports can be viewed for information about its establishment, which includes the survey components, the participatory approach, values and principles, governance, sample design (including study size), promotional campaign, data collection steps, results, processing, analysis and publishing, as well as challenges encountered [25]. Briefly, the target population was composed of all permanent residents of Nunavik, aged 16 years and more, on the James Bay and Northern Quebec Agreement registry of beneficiaries. A non-proportional stratified sampling plan was deployed for each community to obtain reliable estimates for the two age groups (“16 to 30 years old” and “31 years old and more”). A simple random sample without replacement was planned for the two age groups, in addition to the Q2004 participants that were invited to directly integrate Q2017. It was anticipated to survey 2,000 individuals (1,000 for each age group as the target sample size), but a total of 1326 Nunavimmiut aged ≥ 16 years participated with an overall response rate of 30.7% for the 16–30 age group and of 41.5% for the ≥ 31 years age group [25]. All Q2017 participants were informed of their clinical test results, which were returned to their medical chart, and instructions for follow-up with local health services were communicated, when needed [25].

### Data sources, variables and measurements

The following Q2017 databases were exploited for this project: 1) the laboratory results, since all Q2017 participants were invited to submit blood and stool samples; 2) the sociodemographic, psychosocial, and physical health and food security questionnaires answers and 3) the medical chart review performed by trained nurses [25].

#### Primary *H. pylori* outcomes

The three *H. pylori* primary outcomes were: 1) the presence of antibodies against the bacteria identified with the Biorad Platelia *H. pylori* IgG Assay using an amount of at least 0.2 ml of serum and optical density (OD) results were classified as seronegative (≤ 0.90), equivocal (> 0.90 and < 1.10) and seropositive (≥ 1.10); 2) the presence of bacterial antigens in 100 μL of stools with colonisation defined as a positive result (OD ≥ 0.2), negative (< 0.15) or equivocal (≥ 0.15 and < 0.2) using an amplified sandwich Enzyme Immuno-Assay (IDEIA ^TM^ Hp StAR^TM^, Premier Platinum HpSA, Meridian Diagnostics, Cincinnati, USA); and 3) at least one prior diagnosis of colonisation in the medical chart with: i) the non-mutually exclusive list of tests performed for diagnosis (i.e. UBT, biopsy, rapid urease test [RUT], serology, isotope ratio mass spectrometry [IRMS], pathology report, any combination of tests or unknown); ii) the number of diagnosis; and iii) the years they were recorded.

#### Potential clinical consequences of *H. pylori* colonization

The following variables from the medical chart review were also exploited, i.e. the presence of at least one diagnosis (including the list of diagnostic tests and the years they were recorded) related to: 1) peptic (a synonym of gastro-duodenal) ulcer; 2) gastritis, including the type suspected (i.e. related to alcohol consumption, secondary to medication, viruses) and 3) gastric cancer (i.e. gastric adenocarcinoma or mucosa-associated lymphoid tissue [MALT] lymphoma [27]). Additional variables from the laboratory and questionnaire databases were extracted: 1) the presence of faecal occult blood using a Faecal Immunochemical Test (OC-Auto SENSOR DIANA iFOB) performed on stools of participants aged ≥ 50, based on the recommendation of the Canadian Cancer Society (Polymedco, New York, USA); 2) anaemia for which diagnosis and classification is described by another Q2017 research team [28]; 3) self-reported involuntarily weight loss (“During the last six months, have you involuntarily lost weight and not gained it back? (Did you have to tighten your belt or do your clothes fit more loosely?)”; and 4) self-reported blood in stool (“In the last six months, did you have very black stool (like coal tar) or have you ever seen blood in your stool?”).

#### Other variables

Based on a literature review and preliminary analyses, the following variables were retained and classified in three groups: 1) Human density or contact variables such as administrative coastal region (Hudson or Ungava), community size (small with < 1,000 or large with ≥ 1,000), the number of household members and the presence of “overcrowding” (person per room ratio > 1); 2) Water-related variables such as the main drinking water sources during the last summer and winter, with the modalities regrouped into three categories: i) municipal (regrouping “municipal system tap water/water tank” and “Tap directly at the water plant”); ii) bottled water; and iii) natural (regrouping “from nearby lake, river or stream” and “melted snow, ice or iceberg”) and end-point water treatment (“At home, do you treat the water you drink by any methods? Boiling, Filtering [Brita, charcoal or similar] and “Other types of treatment”). Few discrepancies were observed between the summer and winter main drinking sources, with the highest risk category retained in case of discrepancies (natural > municipal > bottled water). Preliminary analysis using the “presence of a water tank at home” and “when the water tank was cleaned the last time” questions did not yield conclusive results and were excluded from the analysis; and 3) potentially confounding variables such as age (in years), gender (men or women), food insecurity (“secure or marginally insecure”, “moderately” or “severely insecure”), frequency of alcohol drinking (“never”, “less than once a month”, “once to 3 times a month”, “once to 2 times a week”, “3 – 6 times a week” or “daily or almost daily”) and smoking status (“never smoked”, “former smoker”, “occasional smoker” or “daily smoker”) were retained.

### Statistical methods

#### Exclusion criteria and population weights

**Figure 1.**
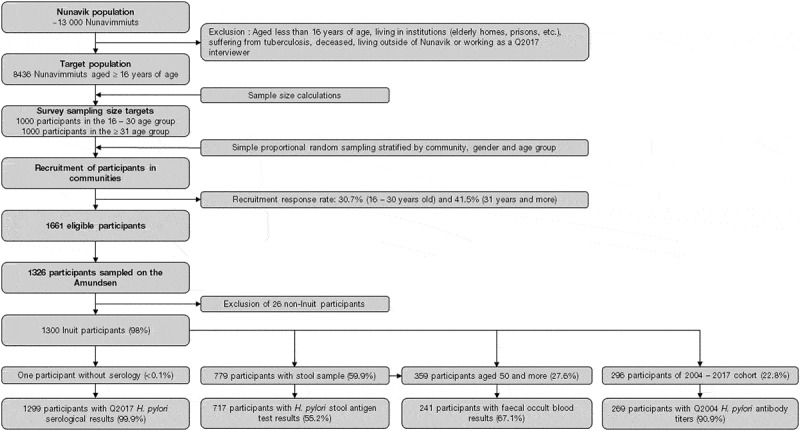
Number of participants at every step of the Q2017 Nunavik Health Survey and for each *H. pylori* primary health outcome and potential clinical consequences.

All non-Inuit participants (*n* = 26) were excluded from the analysis (Figure 1). To harmonise with the aforementioned colorectal cancer methodology, the five Inuit participants aged < 50 years with occult faecal blood testing were excluded from this analysis. Individuals with a gastritis episode recorded in the medical chart as being caused by other etiologic agents (e.g. alcohol [*n* = 39], virus and medication [each *n* < 5]) by the local health professional, were not considered suffering potentially from a *H. pylori-*related stomach inflammation for this analysis. Prevalence represents the proportion of “positive” tests results or the “yes” answers to Q2017 questions on the total sample number, excluding equivocal results and missing data. Equivocal results represented < 5, 17 and 78 participants in regards to the SAT and occult faecal blood tests, Q2017 and Q2004 serologies, respectively. Results associated with a number of cases under five cannot be presented, in order to respect Q2017 participants’ confidentiality and researchers’ agreements. Population weights were available only for some outcomes (*H. pylori* seroprevalence, occult blood, anaemia, self-reported involuntary weight loss and presence of fresh or digested blood in stools). To account for the complex sampling design, a bootstrapped balanced repeated replication approach was used, with 500 iterations to create 95% confidence intervals (CI).

#### Statistical analysis

Modified Poisson regression models with a robust variance estimator were used with generalised estimation equations yielding prevalence ratios (PRs). The first set of regression models used the three main *H. pylori* outcomes (i.e. SAT, serology and prior diagnosis of colonisation in the medical charts) as dependent variables, and the following explanatory (administrative coastal region, community size, the number of household members and the main drinking water sources) and potentially confounding (age and age-squared, gender, food insecurity, frequency of alcohol drinking and smoking status) variables. The last three variables were treated on an ordinal scale, following robust preliminary analysis, with PRs interpreted as a change per level increase. The second set of regression models used each *H. pylori* potential clinical consequences as dependent variables, and *H. pylori* serology and SAT as explanatory variables, adjusted for age. Sensitivity analyses entailed: i) re-estimating seroprevalence models with population weights, ii) replacing the number of household members by overcrowding (following collinearity issues between both variables) and iii) replacing the main water source by end-point water treatment (boiling, filtering or other). Results are accompanied by 95% confidence intervals (95% CI) and *p*-values, and interpreted according to the level of compatibility of data with the null hypothesis (i.e. that the outcomes do not differ according to the levels of each explanatory variable) [29]. All statistical analyses were performed in SAS software, Version 9.4 (SAS Institute Inc., Cary, NC, USA). Using the SAT as the “gold standard”, sensitivity, specificity, as well as positive and negative predictive values for serology, were calculated [30].

## Results

### Description of the studied populations and missing data

The number of participants at every step of the selection process for this project is detailed in Figure 1. Amongst the 1326 Q2017 participants, 1300 declared being Inuit and different sub-samples of them were retained for this project: 1299 had serological results (99.9%), 717 had SAT results (55.2%), 1201 had their medical charts reviewed (92.4%), 241 had faecal occult blood results (67.1% of those aged ≥ 50 years) and 269 had archived serum tested from Q2004 (90.9% of the participants to both Q2004 and Q2017) (Figure 1). The description of the Q2017 sampled populations in regards to basic variables and bivariate analysis is available in the Supplemental Table S1.

### Description of *H. pylori* primary outcomes and potential clinical consequences

Nunavik’s Q2017 population *H. pylori* seroprevalence was estimated at 72.5% (95% CI, 69.9; 75.0) while the unweighted seroprevalence from Q2004 was 76.6% (95% CI, 71.1; 81.3) (Table 1). In the 717 participants that submitted stools, the unweighted prevalence of colonisation was 70.9% (95% CI, 67.4; 74.1) (Table 1). The estimated population prevalence of occult faecal blood was 12.7% (95% CI, 8.2; 19.3) among individuals aged ≥ 50 (Table 1). An estimated 19.6% (95% CI, 17.3; 22.2) of the population had signs of anaemia either classified as caused by iron deficiency (9.1%, 95% CI, 7.6; 11.0), chronic inflammation (3.9%, 95% CI, 2.7; 5.5) or unexplained (6.6%, 95% CI, 5.3; 8.3) (Table 1). The estimated population prevalence of self-reported symptoms in the year before Q2017 was 22.3% with involuntary weight loss (95% CI, 19.7; 25.2) and 13.3% with fresh or digested blood in stools (95% CI, 11.1; 15.7) (Table 1).

**Table 1.** Description of all *H. pylori* related health outcomes using the Q2004 and Q2017 Inuit Health Survey.

Outcome variables			Total n	Positive or “Yes”		Negative or “No”	
				n	Prevalence (95% CI)	n	Prevalence (95% CI)
***Helicobacter pylori*****primary health outcomes**							
	Q2004 unweighted *H. pylori* serology		269	206	76.6 (71.1; 81.3)	46	17.1 (13.0; 22.1)
	Q2017 unweighted *H. pylori* stool antigen test		717	508	70.9 (67.4; 74.1)	206	28.7 (25.5; 32.2)
	Q2017 unweighted medical history of *H. pylori* colonisation		1201	341	28.4 (25.9; 31.0)	860	71.6 (69.0; 74.1)
	Q2017 weighted *H. pylori* serology		1299	893	72.5 (69.9; 75.0)^a^	328	22.4 (20.0; 25.0)
***Helicobacter pylori*****potential clinical consequences**^**i**^							
	Q2017 weighted occult faecal blood		241	213	12.7 (8.2; 19.3)^b^	24	86.4 (79.9; 91.1)
	Q2017 weighted prevalence of anaemia		1300	292	19.6 (17.3; 22.2)^c^	1008	80.4 (77.8; 82.7)
		Q2017 weighted prevalence of iron deficiency anaemia	1299	137	9.1 (7.6; 11.0)^d^	1162	90.9 (89.0; 92.4)
		Q2017 weighted prevalence of chronic inflammation anaemia	1299	53	3.9 (2.7; 5.5)^e^	1246	96.1 (94.5; 97.3)
		Q2017 weighted prevalence of unexplained anaemia	1299	102	6.6 (5.3; 8.3)^f^	1197	93.4 (91.7; 94.7)
	Q2017 weighted involuntary weight loss		1258	285	22.3 (19.7; 25.2)^g^	973	77.7 (74.8; 80.3)
	Q2017 weighted presence of fresh or digested blood in stools		1261	153	13.3 (11.1; 15.7)^h^	1108	86.7 (84.3; 88.9)
	Q2017 unweighted medical history of gastritis		1201	135	11.2 (9.6; 13.2)	1066	88.8 (86.8; 90.4)
	Q2017 unweighted medical history of peptic ulcer		1201	29	2.4 (1.7; 3.5)	1172	97.6 (96.5; 98.3)

^a^Q2017 unweighted *H. pylori* serology = 68.7% (95% CI, 66.2; 71.2); ^b^Q2017 unweighted occult faecal blood = 10.0% (95% CI, 6.7; 14.5); ^c^Q2017 unweighted presence of anaemia = 22.5% (95%CI, 20.3; 24.8); ^d^Q2017 unweighted prevalence of iron deficiency anaemia = 10.5% (95% CI, 9.0; 12.3); ^e^Q2017 unweighted prevalence of chronic inflammation anaemia = 4.1% (95% CI, 3.1; 5.3); ^f^Q2017 unweighted prevalence of unexplained anaemia = 7.9% (95% CI, 6.5; 9.4); ^g^Q2017 unweighted involuntary weight loss = 22.7% (95% CI, 20.4; 25.1); ^h^Q2017 unweighted presence of black stools or blood in stools = 12.1% (95% CI, 10.4; 14.1); ^i^The number of gastric cancer and its prevalence are not presented because of cases < 5.

The 1201 reviewed medical charts indicated that 28.4% (95% CI, 25.9; 31.0) of participants had at least one prior diagnosis of *H. pylori* colonisation (Table 1). Of the 341 medical charts with a prior *H. pylori* diagnosis, episodes were reported: once (91.2%), twice (5.0%), three- (1.5%), four- (1.2%), five- (0.6%) and six-times (0.6%). A wide variety of non-mutually exclusive diagnostic methods were reported: Unknown test (32.6%), UBT (29.3%), biopsy (22.3% of which 26.3% using RUT and 33.3% with a pathology report), serology (13.8%) and IRMS (10.6%). Gastritis potentially associated with *H. pylori* was reported in 11.2% (95% CI, 9.6; 13.2) of the 1201 medical charts (Table 1), with episodes reported once (92.6%), twice (6.7%) and four-times (0.7%). Peptic ulcers were reported in 2.4% (95% CI, 1.7; 3.5) of reviewed medical charts (Table 1), with < 5 participants reporting two episodes. Few records reported diagnostic methods for gastritis and peptic ulcers, such as endoscopy and biopsy. Less than five medical charts reported a history of gastric-related cancer (carcinoma), with the exact number and prevalence that cannot be reported due to Q2017 confidentiality agreements.

### Variables associated with *H. pylori* primary outcomes

**Figure 2.**
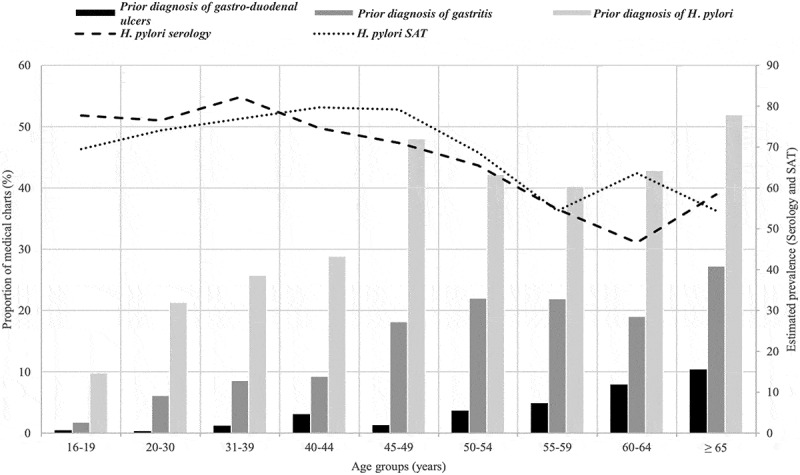
Estimated age groups prevalences of *H. pylori* serology, SAT, and at least one prior diagnosis of colonisation, gastritis and peptic ulcers in the medical charts.

Seropositivity was more frequent among inhabitants of the Hudson Coast (Prevalence ratio [PR] = 1.15, 95% CI, 1.07; 1.25) compared to Ungava Coast (Table 2). Seroprevalence in those drinking from municipal water (0.87, 95% CI, 0.78; 0.99) was lower compared to those drinking mainly bottled water (Table 2). As for potential confounding variables, seroprevalence was associated with age (Table 2 and Figure 2 to observe the quadratic relationship) and higher for men (PR = 1.11, 95% CI, 1.03; 1.19) compared to women (Table 2). Increasing alcohol drinking was inversely correlated with the presence of antibodies (PR = 0.97, 95% CI, 0.95; 1.00). All other estimates were compatible with the null value (i.e. community size, each additional household member, natural versus municipal and bottled water, increasing food insecurity and smoking status) (Table 2).

**Table 2.** Prevalence ratio (PR) and 95% confidence intervals (95% CI) of the multivariable analysis for each *H. pylori* primary outcome.

Variables		Seroprevalence		Prevalence of colonisation		Prevalence of at least one prior diagnosis in the medical chart	
		PR (95% CI)	*p*-value	PR (95% CI)	*p*-value	PR (95% CI)	*p*-value
**Human density or contact variables**							
	Hudson versus Ungava Coast^a^	1.15 (1.07; 1.25)	0.0002	1.11 (0.99; 1.23)	0.07	0.92 (0.72; 1.17)	0.5
	Larger versus small community^b^	0.99 (0.92; 1.07)	0.9	1.06 (0.94; 1.20)	0.3	0.81 (0.63; 1.05)	0.1
	Each additional household member	1.01 (0.99; 1.02)	0.3	1.03 (1.01; 1.05)	0.01	1.00 (0.94; 1.06)	0.09
**Water related variables**							
	Municipal versus bottled water	0.87 (0.78; 0.99)	0.03	0.84 (0.72; 0.98)	0.03	0.58 (0.35; 0.96)	0.03
	Natural versus bottled water	0.88 (0.76; 1.02)	0.09	0.72 (0.57; 0.92)	0.008	0.67 (0.39; 1.18)	0.2
	Natural versus municipal water	1.01 (0.91; 1.12)	0.9	0.86 (0.70; 1.04)	0.1	1.17 (0.86; 1.57)	0.3
**Potential confounding variables**							
	Age	1.02 (1.00; 1.03)	0.02	1.03 (1.01; 1.05)	0.005	1.05 (1.01; 1.09)	0.02
	Squared age	1.00 (1.00; 1.00)	0.002	1.00 (1.00; 1.00)	0.004	1.00 (1.00; 1.00)	0.3
	Men versus women	1.11 (1.03; 1.19)	0.004	1.05 (0.95; 1.16)	0.3	0.68 (0.52; 0.89)	0.005
	Increasing food security	1.02 (0.96; 1.07)	0.5	1.07 (0.99; 1.15)	0.1	1.15 (0.97; 1.36)	0.1
	Increasing alcohol drinking	0.97 (0.95; 1.00)	0.05	0.96 (0.92; 1.00)	0.04	1.00 (0.91; 1.10)	1.0
	Smoking status	1.00 (0.95; 1.04)	0.9	1.01 (0.95; 1.07)	0.8	1.10 (0.97; 1.25)	0.1

^a^Administrative coastal region; ^b^Small with < 1,000 or large with ≥ 1,000.

SAT prevalence increased by 3% for each additional household member (PR = 1.03, 95% CI, 1.01; 1.05), was lower in those drinking municipal water (PR = 0.84, 95% CI, 0.72; 0.98) or natural water (PR = 0.72, 95% CI, 0.57; 0.92) compared to bottled water, followed a non-linear relationship with age similar to the one for seroprevalence (Figure 2) and decreased by 4% for each incremental change in the frequency of alcohol drinking (PR = 0.96, 95% CI, 0.92; 1.00) (Table 2). All other estimates were compatible with the null value (i.e. administrative coastal region, community size, drinking natural versus municipal and bottled water, gender, increasing food insecurity and smoking status) (Table 2).

Estimates for the model using prevalence of at least one prior *H. pylori* diagnosis in the medical chart were decreased for participants mainly drinking municipal water compared to bottled water (0.58, 95% CI. 0.35; 0.96) (Table 2). Estimates were only linearly associated with age and inverted for gender, as prevalence was lower for men (0.68, 95% CI, 0.52; 0.89) compared to women (Table 2). All other estimates were compatible with the null hypothesis (Table 2).

All sensitivity analyses produced estimates similar to the main results except that increasing alcohol drinking was closer to the null value in the weighted serology models (Supplementary Tables S2, S3 and S4) and that prevalence of prior diagnosis in medical charts was increased for participants living in “overcrowding” conditions (PR = 1.27, 95% CI, 1.03; 1.57) (Supplementary Table S3).

### Variables associated with *H. pylori* potential clinical consequences

The prevalence of faecal occult blood was higher in SAT-positive (PR = 2.76, 95% CI, 1.00; 7.61) compared to SAT-negative participants aged ≥ 50, while the estimate with serology was compatible with the null value (Table 3). Prevalence for “mild, moderate and severe” anaemia, as well as for each type (i.e. iron-deficiency, chronic or unexplained) was similar across *H. pylori* (serology and SAT) results, with all estimates compatible with the null value (Table 3). No difference in the proportion of participants self-reporting involuntary weight loss or “black stools or blood in stools” according to *H. pylori* status was observed (Table 3). The prevalence of at least one prior *H. pylori* diagnosis in the medical chart was lower in participants with a positive serology (PR = 0.80, 95% CI, 0.66; 0.96) and positive SAT (0.49, 95% CI, 0.39; 0.62) results (Table 3). The prevalence of at least one prior gastritis diagnosis in the medical chart was also lower for SAT-positive participants (PR = 0.39, 95% CI, 0.26; 0.59), but similar for serology results (Table 3). Prevalence of at least one prior diagnosis of peptic ulcers was similar across *H. pylori* test results (Table 3). In addition, individuals aged ≥ 50 who had positive faecal occult blood results self-reported more frequently the presence of fresh or digested blood in their stool (PR = 2.57, 95% CI, 1.10; 6.01), while none suffered from anaemia (results not shown).

**Table 3.** Multivariable associations between potential *H. pylori* consequences (outcomes) and, *H. pylori* antibody seroprevalence and prevalence of colonisation (explanatory variables).

*H. pylori* potential consequences (outcomes)		n	*H. pylori* serology+^a^ (95% CI ^b^)	Prevalence ratio (95% CI)	*p*-value	*H. pylori* SAT+^c^ (95% CI)	Prevalence ratio (95% CI)	*p*-value
Occult faecal blood^d^								
	No	201	8.9 (3.7; 19.8)	Ref.		5.2 (1.95; 13.1)	Ref.	
	Yes	20	14.1 (7.8; 24.3)	1.38 (0.55; 3.31)	0.4	15.0 (9.5; 23.0)	2.76 (1.00; 7.61)	0.05
Mild, moderate, or severe anaemia								
	No	947	19.9 (15.6; 24.9)	Ref.		27.7 (22.0; 34.2)	Ref.	
	Yes	274	19.8 (17.0; 22.8)	1.10 (0.86; 1.40)	0.4	19.3 (16.1; 23.0)	0.81 (0.60; 1.08)	0.2
Iron-deficiency anaemia								
	No	1089	6.9 (4.7; 10.1)	Ref.		10.2 (6.7; 15.1)	Ref.	
	Yes	132	10.0 (8.2; 12.3)	1.37 (0.90; 2.07)	0.1	9.1 (6.8; 11.9)	0.86 (0.52; 1.41)	0.5
Chronic inflammation anaemia								
	No	1172	4.3 (2.5; 7.2)	Ref.		5.8 (3.3; 10.0)	Ref.	
	Yes	49	3.1 (2.1; 4.7)	1.07 (0.63; 1.82)	0.8	3.5 (2.2; 5.6)	0.87 (0.41; 1.86)	0.7
Unexplained anaemia								
	No	1128	8.7 (6.0; 12.3)	Ref.		11.7 (7.9; 16.8)	Ref.	
	Yes	93	6.7 (5.1; 8.7)	0.89 (0.59; 1.36)	0.6	6.7 (4.8; 9.2)	0.74 (0.44; 1.23)	0.2
Self-reported involuntary weight loss								
	No	909	19.6 (15.6; 24.3)	Ref.		23.6 (18.2; 30.0)	Ref.	
	Yes	272	23.6 (20.4; 27.2)	1.07 (0.83; 1.36)	0.6	21.5 (18.1;25.3)	0.92 (0.68; 1.25)	0.6
Self-reported presence of black stools or blood in stools								
	No	1036	14.1 (9.8; 19.9)	Ref.		15.0 (10.7; 20.7)	Ref.	
	Yes	148	13.5 (10.9; 16.6)	0.95 (0.62 1.45)	0.8	12.5 (9.9; 15.7)	0.73 (0.49; 1.08)	0.2
At least one prior diagnosis *H. pylori* colonisation in the medical chart								
	No	819	37.9 (32.7; 43.5)	Ref.		44.3 (37.5; 51.4)	Ref.	
	Yes	311	24.7 (21.8; 27.7)	0.80 (0.66; 0.96)	0.02	21.2 (17.7; 25.2)	0.49 (0.39; 0.62)	< 0.0001
At least one prior diagnosis of gastritis in the medical chart								
	No	1066	14.5 (11.0; 18.8)	Ref.		21.1 (15.9; 27.5)	Ref.	
	Yes	135	9.5 (7.7; 11.7)	0.94 (0.67; 1.32)	0.7	7.4 (5.3; 10.2)	0.39 (0.26; 0.59)	< 0.0001
At least one prior diagnosis of peptic ulcer in the medical chart								
	No	1102	3.9 (2.2; 6.7)	Ref.		2.6 (1.1; 6.1)	Ref.	
	Yes	28	2.0 (1.2; 3.2)	0.73 (0.35; 1.54)	0.4	1.7 (0.9; 3.5)	0.87 (0.28; 2.73)	0.8

^a^Weighted seroprevalence; ^b^CI: Confidence interval; ^c^Unweighted prevalence; ^d^Models adjusted for age only because of smaller sample size.

### Comparisons between *H. pylori* SAT and serological tests

In the 688 Q2017 participants who had simultaneously *H. pylori* SAT and serology results, sensitivity and specificity values of the serological test were, respectively, 0.85 (95% CI, 0.82; 0.88) and 0.67 (95% CI, 0.60; 0.74) compared to the detection of antigens in the stools, while positive and negative predictive values were, respectively, 0.86 (95% CI, 0.83; 0.89) and 0.66 (95% CI, 0.59; 0.72) (Table 4).

**Table 4.** Comparisons of *Helicobacter pylori* serological results in the sample compared to the stool antigen test used as the gold standard.

Serological results	Stool antigen test (SAT)		Total
	Positive	Negative	
Seropositive	True-positives (TP) 415	False-positives (FP) 66	481 (Serology+)
Seronegative	False-negatives (FN) 71	True-negatives (TN) 136	207 (Serology-)
Total	486 (SAT+)	202 (SAT-)	688^a^

^a^This represents the sample size of Q2017 participants who had simultaneously *H. pylori* serological and SAT results, excluding all equivocal or undetermined results.

Sensitivity=TP/SAT+=415/486=0.85

Specificity=TN/SAT−=136/202=0.67

Positive predictive value=TP/Serology+=415/481=0.86

Negative predictive value=TN/Serology−=136/207=0.66

## Discussion

This is the first large-scale survey aimed at understanding the epidemiology of *H. pylori* in Nunavik. As suspected by local health authorities, bacterial colonisation is very common in Nunavik people (~71%) and seroprevalence data suggest that exposure levels remained stable between 2004 and 2017. The only previous report of *H. pylori* in Nunavik dates back to 1998, where 27% of the 100 randomly selected umbilical cords tested positive (*n* = 8) or equivocal (*n* = 19) for anti-*H. pylori* IgG antibodies [31]. This sample was composed of Inuit women of all ages, but mainly those under 25. The Q2017 prevalence using similar age groups of men and women remains quite elevated (~75% for the “16-19” and “20-30 years old” age groups in Figure 2) compared to Hodgins and his collaborators [31]. The Q2017 prevalences also remain elevated compared to southern Quebec (~30% in 236 subjects aged 20 to 74 years old) [32] or the rest of Canada (13–30% in adult patients) [33–35], but comparable to other Arctic indigenous communities [17,36–41].

Approximately 30% of participants sought medical attention for *H. pylori*, with almost half of those aged ≥ 60 with at least one prior diagnosis of *H. pylori* colonisation in their medical charts. Successful treatment of these individuals could explain the decreasing prevalence trend (serology and SAT) observed in the older age groups (Figure 2) and this hypothesis is also supported by lower prevalences for both outcomes in participants with a prior colonisation diagnosis in their medical charts. However, linking past diagnoses/treatment(s) and confirmation of eradication using the medical chart database with Q2017 *H. pylori* outcomes, was not possible. Figure 2 also supports visually that chronicity of the infection leads to increasing symptomatology for gastritis and peptic ulcers, as people age, but this remains to be demonstrated. The prevalence of gastritis (11.2%) and peptic ulcers (2.4%) estimated in Q2017 (using multiple diagnostic methods) was similar to what was observed by endoscopic assessment in western Canadian Arctic communities. Fagan-Garcia and colleagues (2019) estimated that 12–15% of their 309 participants (from Yukon and Northwest Territories [NT]) had gastritis while 3–4% had gastric ulcers. However, histopathology results showed to be “strikingly different” from endoscopy and a prevalence of 72.4% for chronic gastritis (mild to severe compared to none) was observed, with an occurrence of 99% of *H. pylori*-positive compared to 13% in negative participants (χ^2^ *p*-value ≤ 0.0001) [39]. Frequent histopathological gastritis in *H. pylori*-positive biopsy participants (i.e. 94% for acute and 100% for chronic gastritis) from Aklavik (NT) was also observed by Cheung and colleagues (2014) [41].

Less than five cases (the exact number cannot be revealed) of past gastric cancer associated with *H. pylori* colonisation were recorded amongst the 1201 medical chart reviews. Using Q2017 to capture this information is not recommended, as past and contemporaneous Nunavimmiut dealing with gastric cancer are less likely to be captured or to participate in this type of survey (e.g. mortality, treatment). Exploring Quebec’s Cancer Registry is preferred, but specific details concerning the number of gastric cancers affecting Nunavimmiut are lacking, as numbers below 5 are not presented or merged within the “others” cancer categories for the same confidentiality reasons [42]. Based on the 2017 incidence of gastric cancer for Quebec (estimated at 0.11 per 1000 persons-years, since 890 cases occurred for a population of 8.3 M) and Nunavik’s population estimated at 12 488 inhabitants [25], it would be expected that ~ 1.4 individuals would receive this type of diagnosis yearly. However, this estimation is flawed and the true incidence remains unknown for two reasons: 1) Nunavik’s population is predominantly younger [43] and gastric cancer incidence rates are lower for these age groups [44] expecting to decrease the annual expected number, but 2) since *H. pylori* is a recognised carcinogen, elevated *H. pylori* colonisation prevalence, as supported by Q2017, compared to the south could increase the expected number. Furthermore, the impact of *H. pylori* strains carrying the cytotoxin-associated gene (cagA) or any other virulence factor [45,46], which increases the risk of colonisation and the development of peptic ulcers (gastric and duodenal) and gastric cancer, is yet to be documented in this region. Higher rates of gastric cancer are observed in other Arctic populations [37,47,48]. Therefore, estimating an age-standardised incidence of gastric cancer in Nunavik would be of great value. This is a crucial determinant of the potential benefits of intensive test-and-treat recommendations, and whether they outweigh the harms of such an approach in a very high-endemicity region.

Faecal occult blood was detected in 12.7% of participants aged ≥ 50 and was correlated with colonisation and self-observing fresh or digested blood in stools. Bleeding peptic ulcers associated with *H. pylori* colonisation have been reported in the Q2017 medical chart review. In the literature, individuals with combined positive faecal occult blood and *H. pylori* RUT test were more likely to have upper GI endoscopic findings [49]. However, other sources of blood loss (haemorrhoids, anal fissures, inflammatory bowel diseases, diverticulitis, cancer, etc.) [50] cannot be excluded and only the medical follow-up for this test will help understand the potential causes associated with this bleeding.

Mild, moderate, or severe anaemia was diagnosed in 19.6% of Nunavimmiut and absence of correlations between each type of anaemia and *H. pylori* outcomes (serology or SAT) were observed. This suggests the bacteria could be a clinically minor factor in anaemia in Nunavik adults, but the introduction of a temporal or confounding bias cannot be excluded. The Q2017 anaemia component revealed that iron-deficiency anaemia was highly correlated with being a woman of reproductive/childbearing age (16 to 49 years), while other types of anaemia (chronic inflammation and unexplained) were more frequently observed in men and women aged 50 years and more [51]. In addition, an increased likelihood of being *H. pylori* seropositive in women with iron-deficiency anaemia (OR = 2.18, 95% CI, 1.13; 4.23) was observed [51], which differs from our result that is compatible with an absence of association. Both multivariable analyses yield different results, because the population and co-variable adjustments differ (i.e. age, D vitamin, blood mercury and selenium levels, hs-CRP and RBC for Lavoie et al. 2024 and age, region and gender for this project) [51]. These results add to the inconsistent literature on the subject regarding other indigenous communities [40,52–57]. A systematic review and meta-analysis also supported that *H. pylori* positive individuals showed increased likelihood of iron-deficiency anaemia (IDA), iron deficiency and anaemia [58]. *H. pylori* colonisation has been linked to IDA through biologically plausible mechanisms (i.e. low acid levels reducing iron absorption, bacterial competition for iron, iron loss) [59], but the underlying pathophysiology still needs to be clarified [60]. In addition, the 2022 International *H. pylori* management guidelines recommend eradication in patients with unexplained IDA [4]. Our findings of a lack of clear association between IDA and *H. pylori* colonisation add to those in other indigenous communities suggesting that this relationship is setting-specific. Moreover, few data provide evidence that treatment of *H. pylori* among colonised individuals with IDA who are otherwise asymptomatic leads to resolution of anaemia [61,62]. Overall, there is significant uncertainty about whether screening for *H. pylori* on the basis of IDA alone provides a net clinical benefit in Nunavik. This should be investigated in the future.

The variables associated with *H. pylori* colonisation were the administrative coastal region and each additional household member, representing potential proxies of human densities and contact rates, while prevalence did not vary according to “overcrowding” and “community size”. Seroprevalence was also higher in participants from the Hudson, compared to the Ungava Coast, while the proportion of at least one prior diagnosis of colonisation in the medical chart was similar across coasts. The Hudson Coast is more populated than the Ungava Coast, but the observed difference in prevalence between regions may also be caused by other plausible explanations; this should be investigated more formally. In the literature, *H. pylori* prevalence varies at many geographical scales [5,40,52], but is also similar between regions [38,63], with household clustering variables (i.e. overcrowding, number of children, having infected household members) identified as risk factors [40,52,64,65] and sometimes not playing this role [36,63]. Since the number of household members is used to calculate the “overcrowding” variable which represents the person per room ratio, it remains counterintuitive to obtain different results. However, using the numerical variable (i.e. number of household members) should always be privileged to obtain better statistical power and precision [66].

Our results also suggest that the main source of drinking water reported as bottled water could be associated with an increased risk of carrying *H. pylori*. Because contamination of bottled water is unlikely in Canada, sharing water bottles may possibly explain the small increased risk we observed. Moreover, consumption of bottled water is relatively rare in the region (~7%). Therefore, it could not explain by itself the very high prevalence of colonisation. In addition, our results do not support that people acquire the bacteria through natural or municipal drinking sources, if it was to be unexpectedly found in raw waters because of faecal contamination. *H. pylori* DNA was detected in water samples from a water delivery truck and from two lakes samples near a Nunavut community [63]. However, it must be highlighted that municipal water treatments employed in Nunavik (i.e. chlorination, ultra-violets, and reverse osmosis) [67–69], as well as end-point treatment (boiling or filtering with membranes blocking bacteria) inactivate or capture *H. pylori* [70,71]. *H. pylori* prevalence did not vary according to “end-point water treatment” nor “the frequency of cleaning the water tank”. In the literature, *H. pylori* prevalence in indigenous populations can be higher when using “unregulated water” [40] or lower when drinking untreated water [65], supporting inconsistent results. Another hypothesis is that Nunavimmiut reporting drinking bottled water as their main source could lack water in their house more often, which could be confounded by other variables (i.e. the number of household members/overcrowding), but this should be further investigated. It is also possible that the newly created variable combining the last winter and summer main water drinking source may not represent the historical water-related risk with *H. pylori*, as it covers approximately 6 months in the year before Q2017.

Age remains the most consistent variable associated with *H. pylori* outcomes (serology, SAT and at least one prior diagnosis in the medical charts), with peaks of colonisation in the 41–49 age group, like other projects studying adults in other Arctic communities [39,40]. Even if acquisition mainly occurs during childhood, prevalence is higher in adults because of a cohort effect [14,40,72,73]. An association with age is not always observed in other studies [36,63,65]. Prevalence according to gender yielded inconsistent results across *H. pylori* outcome, supporting an increased prevalence for men with the serology, no association with SAT and a decreased proportion of at least one prior diagnosis in women’s medical charts. Inconsistent results with gender are present amongst other indigenous populations [36,39,41,63,65,74]. We also observed a modest protective association of the increased frequency of alcohol consumption in our main results but with discrepancies obtained in some of our sensitivity analyses. Drinking alcohol may offer some antimicrobial protection but this study adds to the inconsistent associations reported in the literature [41,63]. Moreover, it should be emphasised that alcohol consumption remains by itself a risk factor for gastric cancer [75,76]. No association between the *H. pylori* outcomes and smoking status was observed throughout the main and sensitivity analyses, which was also the case in the study realised by McKeown et al. [62]. *H. pylori* prevalence was not associated with levels of food insecurity, a variable privileged over the other socio-demographics characteristics (e.g. employment status, education and income level) [77].

Though UBT is considered the most accurate non-invasive technique for *H. pylori* diagnosis [4,26], logistical challenges and time constraint associated with Q2017 led us to use more convenient mass-screening tools. SAT was proposed with apprehensions that some participants would be reluctant to return stool samples [78]. Serology testing was chosen to allow comparisons between serum from Q2017 and Q2004 (archived), but also because this non-invasive diagnostic test was still recommended while planning Q2017 [79]. For clinical management, SAT results should be prioritised over serology ones, but this is only possible for Q2017 participants that provided stools, representing 55.2% of the 1300 Inuit participants. This low rate of participation may have introduced a selection bias in the analyses [29], since no correction by population weights was possible with this outcome. Even if serology results were not compared to a gold standard during Q2017, as suggested by the Maastricht V/Florence Consensus Report [4], they remain the only *H. pylori* outcome measured for the other Q2017 participants (i.e. 44.8% that did not provide stools). The introduction of a selection bias with serology results is less likely with a 99.9% participation rate. A Cochrane Systematic review about non-invasive tests for *H. pylori* infection suggests that SAT and serology have similar accuracy when compared to the histopathological reference standard [26]. Sensitivity with a fixed specificity of 0.90 was estimated at 0.83 (95% CI, 0.73; 0.90) for SAT and at 0.84 (95% CI, 0.74; 0.91) for serology; in comparison, UBT was estimated at 0.94 and 0.92 when using, respectively, Carbone 13 or 14 isotopes [26]. Al Ofairi and colleagues (2024) suggested that both tests (SAT and serology) may complement each other, with four stages observed: 1) Chronic colonisation (SAT+/serology+); 2) Recent colonisation (SAT+/serology-); 3) Historical colonisation (SAT-/serology+) and 4) non-infected individuals (SAT-/serology-) [80]. However, this doesn’t take into account that imperfect tests are employed. Performing an outcome misclassification bias analysis or employing correction methods could be explored in the future [81,82]. In addition, the serological test used in Nunavik should eventually be validated against invasive diagnostic methods (i.e. histology, culture, PCR) [2]. Because antibodies can persist > 24 months after eradication in most individuals, serology is not recommended to confirm eradication after treatment, which should rather be performed with UBT or SAT [4]. *H. pylori* antibody titres naturally decline in ~ 25% of infected and untreated individuals within a period of 10 years with spontaneous remission occasionally observed [83–85]. Recolonisation is expected to occur in Nunavik as it occurred with other indigenous populations, with a cumulative recolonisation rate of 16% estimated in Alaskan Natives [85]. The similar proportion of participants with antibodies and colonisation in 2017, and between 2004 and 2017 surveys with regards to seroprevalence, suggests that an equilibrium is reached; the rate at which new colonisation/recolonisation/seroconversion occurs is balanced with the rate of bacterial disappearance (following treatment or spontaneous remission) and sero-reversion.

The Q2017 Inuit Health Survey was designed in close collaboration with local communities and stakeholders (i.e. community-driven approach), which significantly improved our knowledge about *H. pylori* burden in Nunavimmiut. The large sample size and the advanced statistical methods (e.g. population weights, sensitivity analysis) employed to decrease potential biases, provides robust results that could be potentially generalised to other Inuit populations. However, the cross-sectional design remains problematic to infer causality, as it is impossible to know whether the measured variables occurred before or after *H. pylori* colonisation [86]. A temporal bias is highly suspected when linking past outcomes observed in the medical chart review with contemporaneous variables and these results should be interpreted with caution. Selection bias may have occurred in analyses based on SAT because multiple participants refused to submit stools; however, adjustment for the unbalanced covariates may have limited this [87]. Despite adjusting for potential confounders found in the literature, residual confounding in the estimates cannot be excluded. Further description and analysis of the medical chart review database was constrained due to significant heterogeneity in reporting diagnostic methods and years of episodes.

As researchers implied in the gastro-intestinal component of Q2017, our role was to provide epidemiological evidence regarding *H. pylori* to inform decisions on clinical guidelines. We voluntarily refrain from making medical recommendations, as this clearly belongs to local communities and public health authorities with the assistance of content-experts. Investigating the burden and impact of *H. pylori* among Inuit of the region is only in its infancy. Nunavik’s Health Authorities can draw from the abundant experience and published literature available from other high-prevalence regions of the Arctic, such as in Alaska and in Nunavut, Yukon and the Northwest Territories, where the Canadian North *Helicobacter pylori* (CANHelp) Working Group (http://www.canhelpworkinggroup.ca/) deploys community-driven projects, of which screening programmes of asymptomatic people. Though the most recent global consensus recommends eradication therapy for all *H. pylori* infected individuals (including the asymptomatic) to prevent gastric cancer [88,89], this approach is not considered realistic or compatible with realities encountered in the Arctic [22,90]. Since 2016, Nunavik’s Health Authorities have rather adapted their strategy to target individuals at higher risk (i.e. dyspeptic patients reporting worrisome systemic symptoms or with family gastric cancer antecedents) [22,23,90]. However, they must remain vigilant by following regional gastric cancer incidence and the rapid evolution of *H. pylori* management guidelines. The net benefits of community-wide eradication must be tested against the numerous potential harms of doing so in communities like those in Nunavik characterised by very high endemicity but with many unknowns (i.e. re-infection rates, treatment success, antimicrobial resistance patterns, treatment compliance, culture safety, etc.) [91,92]. Studies designed to account for all of these are urgently needed.

## Supplementary Material

Supplemental_material.docx

## Data deposition

The SAS program can be requested through the first author. However, the Q2017 data is owned by the Inuit population and a request to access the data needs to be presented to the Q2017 Data Management Committee.
