# Supplementary material for: Increasing our knowledge about the epidemiology of Helicobacter pylori in Nunavik’s Inuit population (Québec, Canada) using Qanuilirpitaa? 2017 cross-sectional survey
Source: Int J Circumpolar Health. 2024 Sep 16;83(1):2398864. doi: 10.1080/22423982.2024.2398864 (PMC11407425; doi:10.1080/22423982.2024.2398864)
Supplement: Supplemental_material.docx [file ZICH_A_2398864_SM7798.docx]

**Supplemental Table 1. Description of Q2017 samples and bivariable analysis associated with the three *H. pylori* primary outcomes, adjusted for age and age^2^**

| **Variables** | | | | ***H. pylori* seroprevalence** | | |  | ***H. pylori* colonization** | | |  |  | **Prior *H. pylori* colonization**  **in medical chart** | | |  |
| --- | --- | --- | --- | --- | --- | --- | --- | --- | --- | --- | --- | --- | --- | --- | --- | --- |
|  |  |  |  | **n** | **Proportion (95% CI)**  **or median (min – max)** | **Prevalence ratio**  **(95% CI; *p*-value) ^a^** |  | **n** | **Proportion (95% CI)**  **or median (min – max)** | **Prevalence ratio**  **(95% CI) ^a^** | ***p*-value** |  | **n** | **Proportion (95% CI)**  **or median (min – max))** | **Prevalence ratio**  **(95% CI) ^a^** | ***p*-value** |
|  | **Increased human density or contacts route** | | | | | |  |  |  |  |  |  |  |  |  |  |
|  |  | Administrative coastal region | |  |  |  |  |  |  |  |  |  |  |  |  |  |
|  |  | Ungava Coast | | 606 | 67.3 (63.5; 71.0) | Ref. |  | 380 | 66.1 (61.1; 70.7) | Ref. |  |  | 617 | 30.3 (26.8; 34.1) | Ref. |  |
|  |  | Hudson Coast | | 615 | 78.9 (75.5; 81.9) | 1.17 (1.09; 1.25) | <0.0001 | 337 | 76.3 (71.4; 80.5) | 1.18 (1.07; 1.31) | 0.001 |  | 584 | 26.4 (22.9; 77.1) | 0.93 (0.78; 1.11) | 0.4 |
|  |  | Community size | |  |  |  |  |  |  |  |  |  |  |  |  |  |
|  |  |  | Small (< 1,000) | 604 | 69.9 (66.1; 73.4) | Ref. |  | 370 | 66.8 (61.8; 71.4) | Ref. |  |  | 648 | 32.1 (28.5; 35.9) | Ref. |  |
|  |  |  | Large (≥ 1,000) | 617 | 76.3 (72.8; 79.5) | 1.08 (1.01; 1.16) | 0.02 | 347 | 75.2 (70.4; 79.5) | 1.11 (1.01; 1.22) | 0.03 |  | 652 | 24.5 (21.2; 28.2) | 0.80 (1.67; 0.96) | 0.01 |
|  |  | Household overcrowding (person per room ratio or PPR) | | | | | | | | | | | | |  |  |
|  |  |  | No (PPR ≤ 1) | 836 | 71.5 (68.4; 74.4) | Ref. |  | 506 | 69.2 (65.0; 73.1) | Ref. |  |  | 828 | 28.0 (25.2; 31.2) | Ref. |  |
|  |  |  | Yes (PPR > 1) | 385 | 76.6 (72.1; 80.6) | 1.02 (0.95; 1.09) | 0.7 | 211 | 74.9 (68.6; 80.3) | 1.04 (0.95; 1.15) | 0.4 |  | 373 | 29.2 (24.8; 31.2) | 1.30 (1.08; 1.57) | 0.006 |
|  |  | Each additional household member | | 1240 | 4 (1; 15) | 1.01 (1.00; 1.03) | 0.08 | 689 | 3.8 (1; 13) | 1.03 (1.01; 1.05) | 0.01 |  | 1145 | 4 (1; 15) | 1.02 (0.99; 1.06) | 0.2 |
|  | **Water transmission route** | | | | | | | | | |  |  |  |  |  |  |
|  |  | Main drinking water source | |  |  |  |  |  |  |  |  |  |  |  |  |  |
|  |  |  | Bottled water | 68 | 83.6 (72.3; 90.9) | Ref. |  | 32 | 81.3 (64.0; 91.3) | Ref. |  |  | 66 | 22.7 (14.2; 34.4) | Ref. |  |
|  |  |  | Municipal system | 919 | 75.8 (72.8; 78.6) | 0.92 (0.82; 1.04) | 0.4 | 542 | 72.0 (68.0; 75.6) | 0.92 (0.76; 1.10) | 0.3 |  | 883 | 27.0 (24.1; 30.0) | 0.95 (0.67; 1.33) | 0.8 |
|  |  |  | Natural sources | 86 | 72.0 (64.1; 78.7) | 0.89 (0.76; 1.02) | 0.2 | 10 | 63.6 (54.4; 72.1) | 0.83 (0.66; 1.05) | 0.09 |  | 187 | 32.1 (25.8; 39.1) | 0.92 (0.63; 1.33) | 0.6 |
|  |  | Water treatment | |  |  |  |  |  |  |  |  |  |  |  |  |  |
|  |  |  | No | 330 | 76.9 (71.9; 81.3) | Ref. |  | 186 | 71.5 (64.6; 77.5) | Ref. |  |  | 301 | 25.9 (21.3; 31.2) | Ref. |  |
|  |  |  | Yes | 897 | 70.9 (67.6; 74.0) | 0.94 (0.88; 1.01) | 0.1 | 497 | 71.0 (66.9; 74.9) | 0.96 (0.86; 1.06) | 0.4 |  | 832 | 28.5 (25.5; 31.7) | 1.01 (0.87; 1.17) | 0.9 |
|  | **Potential confounding variable** | | | | | |  |  |  |  |  |  |  |  |  |  |
|  |  | Gender | |  |  |  |  |  |  |  |  |  |  |  |  |  |
|  |  |  | Women | 801 | 70.7 (67.4; 73.7) | Ref. |  | 480 | 70.2 (66.0; 74.1) | Ref. |  |  | 794 | 30.9 (27.7; 34.2) | Ref. |  |
|  |  |  | Men | 420 | 77.9 (73.6; 81.6) | 1.10 (1.03; 1.19) | 0.004 | 237 | 72.2 (66.1; 77.5) | 1.04 (0.95; 1.15) | 0.4 |  | 407 | 23.6 (19.7; 28.0) | 0.73 (0.60; 0.89) | 0.02 |
|  |  | Food insecurity | |  |  |  |  |  |  |  |  |  |  |  |  |  |
|  |  |  | Secure or marginally insecure | 384 | 69.5 (64.7; 73.9) | Ref. |  | 227 | 66.1 (59.7; 72.0) | Ref. |  |  | 377 | 27.3 (23.1; 32.0) | Ref. |  |
|  |  |  | Moderately insecure | 537 | 75.6 (71.8; 79.1) | 1.06 (0.98.; 1.15) | 0.2 | 336 | 74.4 (69.5; 78.8) | 1.11 (0.99; 1.24) | 0.1 |  | 525 | 26.5 (22.9; 30.4) | 1.08 (0.87; 1.33) | 0.5 |
|  |  |  | Severely insecure | 214 | 75.2 (69.0; 80.6) | 1.07 (0.96; 1.18) | 0.2 | 109 | 70.6 (61.4; 78.4) | 1.06 (0.90; 1.23) | 0.7 |  | 213 | 35.2 (29.1; 41.9) | 1.38 (1.09; 1.76) | 0.008 |
|  |  | Frequency of drinking alcohol | |  |  |  |  |  |  |  |  |  |  |  |  |  |
|  |  |  | Never | 57 | 77.2 (64.6; 86.3) | Ref. |  | 36 | 77.8 (61.4; 88.5) | Ref. |  |  | 51 | 29.4 (18.6; 43.2) | Ref. |  |
|  |  |  | Less than once a month | 158 | 79.7 (72.8; 85.3) | 1.12 (0.90; 1.41) | 0.3 | 86 | 82.6 (73.0; 89.2) | 1.12 (0.90; 1.41) | 0.3 |  | 152 | 25.0 (18.8; 32.5) | 0.79 (0.47; 1.32) | 0.4 |
|  |  |  | Once to 3 times a month | 245 | 78.0 (72.3; 82.7) | 1.21 (1.02; 1.44) | 0.2 | 150 | 77.3 (69.9; 83.3) | 1.21 (1.02; 1.43) | 0.03 |  | 238 | 23.9 (18.9; 29.8) | 0.83 (0.50; 1.37) | 0.5 |
|  |  |  | Once to 2 times a week | 266 | 73.7 (68.1; 78.6) | 1.14 (0.96; 1.34) | 0.3 | 163 | 70.6 (63.1; 77.1) | 1.14 (0.96; 1.34) | 0.1 |  | 266 | 25.6 (20.7; 31.2) | 0.89 (0.55; 1.45) | 0.6 |
|  |  |  | 3–6 times a week | 237 | 72.6 (66.5; 77.9) | 1.03 (0.87; 1.23) | 0.5 | 128 | 63.3 (54.6; 71.2) | 1.03 (0.87; 1.23) | 0.7 |  | 234 | 26.1 (20.8; 32.1) | 0.85 (0.52; 1.38) | 0.5 |
|  |  |  | Daily or almost daily | 185 | 61.6 (54.4; 68.4) | 0.97 (0.80; 1.17) | 0.5 | 115 | 61.7 (52.5; 70.2) | 0.97 (0.80; 1.17) | 0.7 |  | 182 | 39.6 (32.7; 46.8) | 1.03 (0.64; 1.68) | 0.9 |
|  |  | Smoking status | |  |  |  |  |  |  |  |  |  |  |  |  |  |
|  |  |  | Never smoked | 43 | 67.4 (52.2; 79.7) | Ref. |  | 43 | 75.0 (52.0; 89.2) | Ref. |  |  | 41 | 24.4 (13.6; 39.7) | Ref. |  |
|  |  |  | Former smoker | 214 | 67.3 (60.7; 73.2) | 1.04 (0.85; 1.27) | 0.7 | 218 | 65.9 (57.5; 73.4) | 0.98 (0.76; 1.28) | 0.7 |  | 205 | 29.3 (23.4; 35.9) | 1.14 (0.66; 1.97) | 0.6 |
|  |  |  | Occasional smoker | 106 | 75.5 (66.4; 82.7) | 1.00 (0.81; 1.25) | 1.0 | 102 | 69.1 (55.8; 79.9) | 0.94 (0.71; 1.25) | 0.7 |  | 100 | 26.0 (18.3; 35.5) | 1.24 (0.68; 2.27) | 0.5 |
|  |  |  | Daily smoker | 838 | 74.9 (71.9; 77.8) | 1.06 (0.85; 1.33) | 0.6 | 872 | 72.7 (68.6; 76.6) | 0.95 (0.69; 1.29) | 0.9 |  | 833 | 28.1 (25.1; 31.2) | 1.36 (0.81; 2.29) | 0.2 |
| ^a^ Obtained from a robust Poisson regression model adjusted for age and squared-age to obtain linear residues. | | | | | | | | | | | | | | | | |

**Supplemental Table 2. Multivariable sensitivity analysis results of the weighted seroprevalence model**

| **Variables** | | ***H. pylori* seroprevalence**  **ratio (95% CI)** | ***p*-value** |
| --- | --- | --- | --- |
| Human density or contact variables | |  |  |
|  | Hudson versus Ungava Coast | 1.15 (1.06; 1.24) | 0.0005 |
|  | Larger versus small community | 1.02 (0.94; 1.10) | 0.6 |
|  | Each additional household member | 1.01 (0.99; 1.02) | 0.4 |
| Water related variables | | | |
|  | Municipal versus bottled water | 0.88 (0.78; 0.99) | 0.03 |
|  | Natural versus bottled water | 0.89 (0.76; 1.03) | 0.1 |
|  | Natural versus municipal water | 1.01 (0.90; 1.13) | 0.9 |
| Potential confounding variables | |  |  |
|  | Age | 1.02 (1.00; 1.03) | 0.01 |
|  | Squared-age | 1.00 (1.00; 1.00) | 0.002 |
|  | Men versus women | 1.09 (1.02; 1.18) | 0.01 |
|  | Increasing food insecurity | 1.02 (0.96; 1.08) | 0.2 |
|  | Increasing alcohol drinking | 0.99 (0.96; 1.02) | 0.6 |
|  | Smoking status | 1.01 (0.96; 1.05) | 0.8 |

**Supplemental Table 3. Multivariable sensitivity analysis results by replacing “each additional household member” by “overcrowding”**

|  | **Variables** | **Weighted *H. pylori* seroprevalence**  **ratio (95% CI)** | ***p*-value** |  | **Unweighted *H. pylori* seroprevalence**  **ratio (95% CI)** | ***p*-value** |  | **Unweighted *H. pylori* colonization** **prevalence ratio (95% CI)** | ***p*-value** |  | **Unweighted *H. pylori* prior diagnosis**  **prevalence ratio (95% CI)** | ***p*-value** |
| --- | --- | --- | --- | --- | --- | --- | --- | --- | --- | --- | --- | --- |
| Human density or contact variables | | | | | | | | | | | | |
|  | Hudson versus Ungava Coast | 1.15 (1.06; 1.24) | 0.0004 |  | 1.15 (1.07; 1.24) | 0.0003 |  | 1.12 (1.00; 1.25) | 0.05 |  | 0.94 (0.77; 1.16) | 0.6 |
|  | Larger versus small community | 1.02 (0.94; 1.10) | 0.6 |  | 1.00 (0.93; 1.08) | 1.0 |  | 1.05 (0.93; 1.19) | 0.4 |  | 0.81 (0.65; 1.01) | 0.06 |
|  | Overcrowding | 1.04 (0.97; 1.12) | 0.3 |  | 1.02 (0.95; 1.10) | 0.6 |  | 1.07 (0.96; 1.19) | 0.2 |  | 1.27 (1.03; 1.57) | 0.02 |
| Water related variables | | | | | | | | | | | | |
|  | Municipal versus bottled water | 0.88 (0.78; 0.98) | 0.02 |  | 0.87 (0.78; 0.98) | 0.03 |  | 0.83 (0.71; 0.98) | 0.03 |  | 0.72 (0.46; 1.15) | 0.02 |
|  | Natural versus bottled water | 0.89 (0.76; 1.03) | 0.1 |  | 0.89 (0.77; 1.03) | 0.1 |  | 0.72 (0.57; 0.93) | 0.01 |  | 0.80 (0.48; 1.33) | 0.4 |
|  | Natural versus municipal water | 1.01 (0.90; 1.13) | 0.8 |  | 1.02 (0.92; 1.12) | 0.8 |  | 0.88 (0.71; 1.06) | 0.2 |  | 1.11 (0.87; 1.42) | 0.4 |
| Potential confounding variables | | | | | | | | | | | | |
|  | Age | 1.02 (1.00; 1.03) | 0.01 |  | 1.02 (1.00; 1.03) | 0.02 |  | 1.03 (1.01; 1.05) | 0.01 |  | 1.07 (1.04; 1.12) | 0.002 |
|  | Squared-age | 1.00 (1.00; 1.00) | 0.002 |  | 1.00 (1.00; 1.00) | 0.002 |  | 1.00 (1.00; 1.00) | 0.01 |  | 1.00 (1.00; 1.00) | 0.02 |
|  | Men versus women | 1.10 (1.03; 1.19) | 0.006 |  | 1.12 (1.04; 1.20) | 0.002 |  | 1.03 (0.93; 1.14) | 0.6 |  | 0.72 (0.58; 0.90) | 0.003 |
|  | Increasing food insecurity | 1.02 (0.96; 1.08) | 0.5 |  | 1.02 (0.97; 1.07) | 0.5 |  | 1.07 (0.99; 1.16) | 0.09 |  | 1.15 (1.00; 1.31) | 0.05 |
|  | Increasing alcohol drinking | 0.99 (0.96; 1.01) | 0.3 |  | 0.97 (0.95; 1.00) | 0.03 |  | 0.95 (0.91; 0.99) | 0.02 |  | 1.01 (0.94; 1.09) | 0.8 |
|  | Smoking status | 1.01 (0.96; 1.05) | 0.8 |  | 1.00 (0.95; 1.04) | 0.8 |  | 1.00 (0.94; 1.06) | 1.0 |  | 1.09 (0.97; 1.23) | 0.1 |

**Supplemental Table 4. Multivariable sensitivity analysis results by replacing the main water source by the use of end-point water treatment**

|  | **Variables** | **Weighted *H. pylori* seroprevalence**  **ratio (95% CI)** | ***p*-value** |  | **Unweighted *H. pylori* seroprevalence**  **ratio (95% CI)** | ***p*-value** |  | **Unweighted *H. pylori* colonization**  **prevalence ratio (95% CI)** | ***p*-value** |  | **Unweighted *H. pylori* prior diagnosis**  **prevalence ratio (95% CI)** | ***p*-value** |
| --- | --- | --- | --- | --- | --- | --- | --- | --- | --- | --- | --- | --- |
| Human density or contact variables | |  |  |  |  |  |  |  |  |  |  |  |
|  | Hudson versus Ungava Coast | 1.14 (1.06; 1.23) | 0.0009 |  | 1.14 (1.06; 1.23) | 0.0006 |  | 1.10 (0.98; 1.23) | 0.09 |  | 0.97 (0.78; 1.20) | 0.8 |
|  | Larger versus small community | 1.03 (0.95; 1.11) | 0.5 |  | 1.00 (0.93; 1.08) | 1.0 |  | 1.09 (0.97; 1.23) | 0.1 |  | 0.81 (0.66; 1.00) | 0.05 |
|  | Each additional household member | 1.01 (0.99; 1.02) | 0.4 |  | 1.01 (0.99; 1.02) | 0.4 |  | 1.02 (1.00; 1.05) | 0.02 |  | 1.02 (0.98; 1.06) | 0.4 |
| Water related variables | |  |  |  |  |  |  |  |  |  |  |  |
|  | Not using end-point water treatment | 0.96 (0.88; 1.03) | 0.2 |  | 0.96 (0.89; 1.03) | 0.3 |  | 0.99 (0.88; 1.11) | 0.9 |  | 1.11 (0.88; 1.40) | 0.4 |
| Potential confounding variables | |  |  |  |  |  |  |  |  |  |  |  |
|  | Age | 1.02 (1.00; 1.03) | 0.01 |  | 1.02 (1.00; 1.03) | 0.02 |  | 1.03 (1.01; 1.05) | 0.01 |  | 1.07 (1.03; 1.12) | 0.0002 |
|  | Squared-age | 1.00 (1.01; 1.00) | 0.002 |  | 1.00 (1.00; 1.00) | 0.003 |  | 1.00 (1.00; 1.00) | 0.006 |  | 1.00 (1.00; 1.00) | 0.02 |
|  | Men versus women | 1.09 (1.01; 1.17) | 0.02 |  | 1.10 (1.03; 1.18) | 0.007 |  | 1.04 (0.94; 1.15) | 0.4 |  | 0.69 (0.56; 0.87) | 0.001 |
|  | Increasing food insecurity | 1.02 (0.96; 1.08) | 0.6 |  | 1.01 (0.96; 1.07) | 0.6 |  | 1.06 (0.98; 1.15) | 0.2 |  | 1.15 (1.01; 1.32) | 0.04 |
|  | Increasing alcohol drinking | 0.99 (0.96; 1.02) | 0.4 |  | 0.97 (0.95; 1.00) | 0.04 |  | 0.96 (0.92; 1.00) | 0.05 |  | 1.01 (0.94; 1.08) | 0.8 |
|  | Smoking status | 1.01 (0.96; 1.05) | 0.8 |  | 1.00 (0.95; 1.04) | 0.8 |  | 1.01 (0.95; 1.08) | 0.7 |  | 1.09 (0.97; 1.22) | 0.1 |
